# Microstructure and mechanical properties of rostrum in *Cyrtotrachelus longimanus* (Coleoptera: Curculionidae)

**DOI:** 10.1080/19768354.2017.1330764

**Published:** 2017-05-31

**Authors:** Longhai Li, Ce Guo, Xin Li, Shun Xu, Cheng Han

**Affiliations:** a College of Mechanical and Electrical Engineering, Nanjing University of Aeronautics and Astronautics, Nanjing, People’s Republic of China; b Institute of Bio-inspired Structure and Surface Engineering, Nanjing University of Aeronautics and Astronautics, Nanjing, People’s Republic of China; c Key Laboratory of Bionic Engineering (Ministry of Education, China), The College of Biological and Agricultural Engineering, Jilin University at Nanling Campus, Changchun, People’s Republic of China

**Keywords:** Microstructure, mechanical properties, rostrum, lightweight, biomimetic

## Abstract

The microstructure, composition and mechanical properties of the rostrum in *Cyrtotrachelus longimanus* (JHC Fabre) were studied utilizing light, fluorescent, scanning electron microscopy (SEM) and energy-dispersive spectroscopy. SEM images show the morphological characteristics of rostrum’s cross section; it is a typical lightweight multilayer structure – one rigid exocuticle layer and dense endocuticle layers, which construct unevenly overlapping fiber structures. The composition analysis of the rostrum shows that it is mainly composed of C, H, N, O, as well as some metal elements and microelements, such as Mg, Si, Zn, Ca and Na, which contribute to its mechanical performance. The mechanical properties of the rostrum were tested by the electronic universal testing machine, which shows it has high-specific strength and is almost the same as that of the stainless steel. The results may provide a biological template to inspire biomimetic lightweight structure design.

## 1. Introduction

Good mechanical properties of composite materials (high-specific intensity, specific rigidity, damage resistance, etc.) and lightweight structures are very valuable in high-tech industries, such as aerospace, space exploration, express trains, shipping and biomedical applications (Rambo et al. 2006; Sven et al. 2015). In developing superior materials, the common goal is to find structure and composite materials of lighter weight and higher strength (Dai & Yang 2010; Yang et al. 2010). For example, if the weight of a three-stage engine for a missile with a range of 1000 km decreases by 1 kg, the range can increase by 17 km, and if the weight of a bullet decreases by 1 kg, the range increases by 25 km. If the mass of the structure decreases by 1 kg, a spacecraft can save 20 kg in fuel consumption (Yi & Du 2003). However, the traditional metals, polymers and corresponding designs in some high-load, special environments have been unable to meet the requirements for lightweight structures. Natural structures have the desired properties, such as systems with a minimum of material and energy but maximal stability which engineers can use for reference and imitate. Inspired by nature, the synthesis of biomimetic materials, which have excellent properties such as high strength and high modulus, is the target of many researchers.

In nature, many insects have unique skills and gifts and can rapidly adapt to environments, such as ultraviolet radiation from sunlight, high or low temperatures, dry conditions/low humidity, lack of food and external hazards, due to their various multifunctional macro-/micro-lightweight structures, composite biomaterials and appropriate motions (Tabunoki et al. 2016; Zhu et al. 2016).

Many biological structures and materials (such as insect cuticles, mantis shrimp limbs, bone and beetle’s elytra ) have evolved exceptional mechanical properties in spite of the relatively weak material constituents that make up their composition (Vincent & Wegst 2004; Guo et al. 2012; Patek et al. 2013; Porter et al. 2013; Guo et al. 2014). *Cyrtotrachelus longimanus* (JHC Fabre), a major pest of bamboo, classified in the phylum Arthropoda, class Insecta, order Coleoptera and family Curculionidae (Paine et al. 1997; Moon 2015), is a skillful driller and is able to effectively chew in holes and sucks on the bamboo. Its rostrum is an extension of the head of weevils and is often used to bore holes for oviposition (the process of laying eggs) in the host plant tissue (Andrew et al. 2016; Singh et al. 2016). It is a hollow multilayered cylindrical structure, to maximally save materials while ensuring enough strength and stiffness, and this ability has long attracted human beings. However, research on the mechanical properties of rostrum in *C. longimanus* is rare although there are several reports focused on the biological characteristics (Li et al. 2000 ), reproductive system (Wang et al. 2009), reproductive performance (Wang et al. 2005), control strategy (Chen et al. 2005), mouthparts, etc. (Davis 2011).

Bearing the above observation in mind, to meet the requirement of developing lightweight structures and materials, which is always demanded in engineering, the mechanical properties of rostrum in *C. longimanus* were studied. The purpose of this paper is to observe the microstructure of the rostrum in *C. longimanus*, analyze its composition and investigate its mechanical properties. The results may provide biological template to design a new lightweight structure and materials.

## 2. Materials and methods

### 2.1. Scanning electron microscopy (SEM) observation

**Figure 1.**
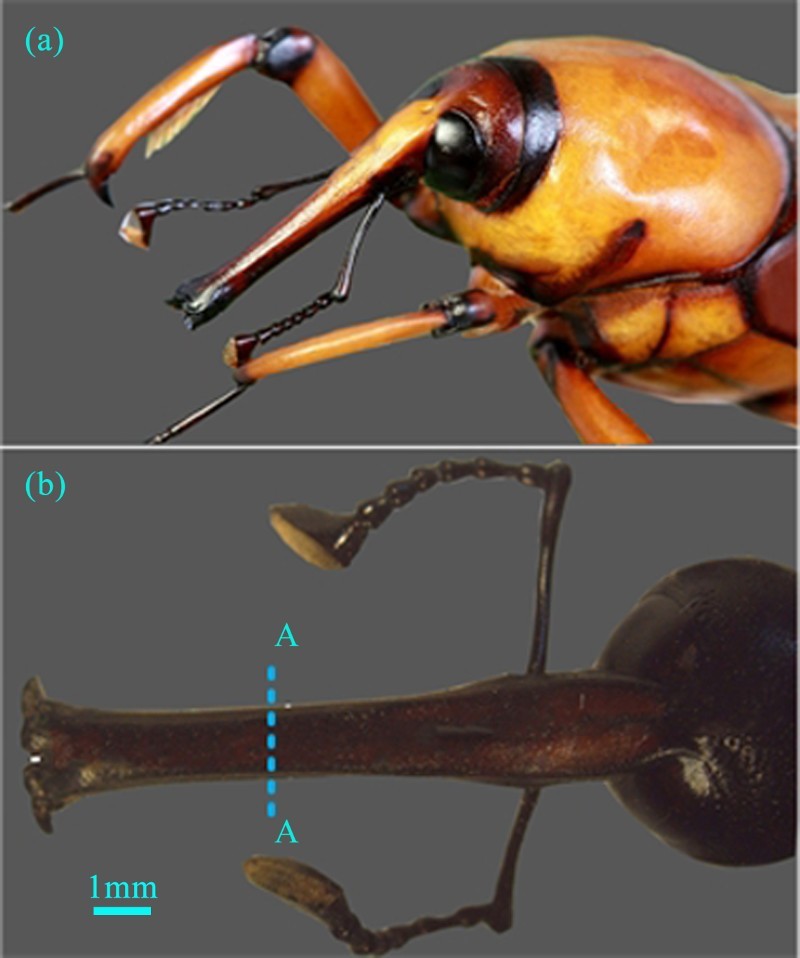
(a) Lateral habitus photograph of the *Cyrtotrachelus longimanus*, (b) lateral view of head of specimen; dashed line indicates approximate location of transverse plane used for SEM.

The specimens used for the investigation were obtained from Leshan, Sichuan Province, China. The SEM experiment and EDS (Energy Disperse Spectroscopy) test were performed at the Institute of Bio-inspired Structure and Surface Engineering, Nanjing University of Aeronautics and Astronautics. Mechanical properties test were performed in Southeast University, Nanjing, China. The tests were kept under a natural light cycle, at a temperature of (25 ± 2)°C and a humidity of 50–70%. Before the observations, some preliminary preparations were done. First, the specimens were cleaned with pure water and air dried for about 30 minutes. Then, the rostrum was dehydrated using different concentrations of alcohol (30%, 50%, 75%, 85% and 95%) for about 40 minutes and air dried for about an hour. A representative specimen was shown in Figure 1(a) and the rostrum was dissected with a fine-edged scalpel (Figure 1(b)). Finally, the rostrum was sputter-coated with gold-palladium alloy and observed using SEM (QUANTA200, FEI, USA) at 20 kV.

### 2.2. Experiment test

**Figure 2.**
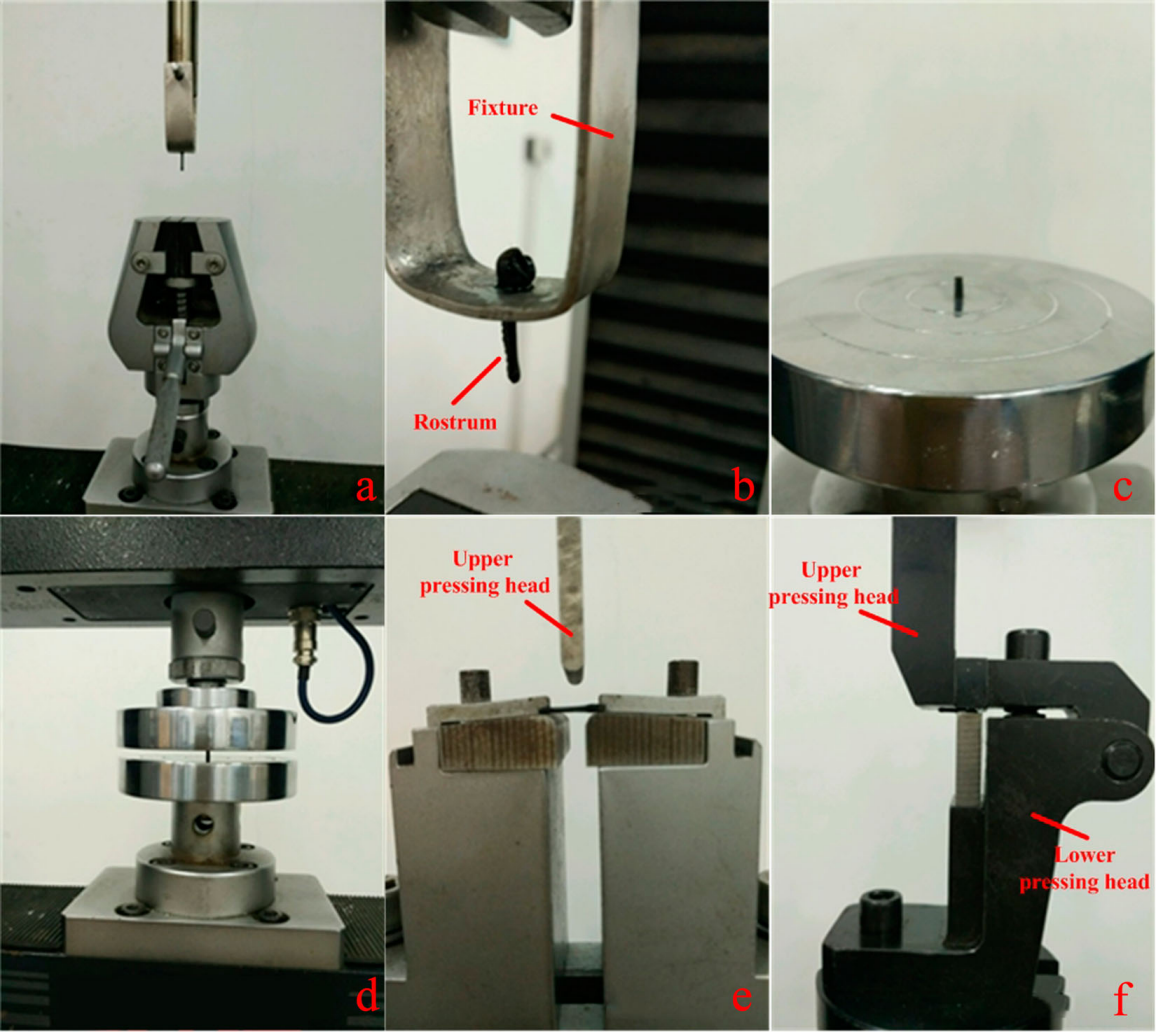
Experiment mechanism.

Mechanical properties’ investigation of the rostrum, including tension (Figure 2(a,b)), compression (Figure 2(c,d)), bending (Figure 2(e)) and shear (Figure 2(f)), was performed by an electronic universal testing machine (CMT4503, China) and the experiment setup was shown in Figure 2. The rostrum of *C. longimanus* ranges from 12 to 15 mm in length, with a diameter of about 0.9 to 1.3 mm. Due to the small size, the fixture of samples for testing was designed specially by ourselves. Previous work has shown that the presence of water in the biological structure affected its mechanical properties. Given the effects in play, the ‘dry’ samples (*N* = 20, each experiment test five samples) and ‘fresh’ samples (*N* = 20, each experiment test five samples) were tested, respectively. ‘Dry’ samples refers to the samples naturally dried for at least one month and ‘fresh’ samples refers to the samples dissected from the insects for no more than an hour. Specifically, the cross section of the rostrum must be polished for compressive test in order to make it flat and other tests without any further processing. The load velocity of 1 mm/minute is set to carry out the tests and the force–displacement curves of the rostrum were obtained.

## 3. Results

### 3.1. Morphological structures

**Figure 3.**
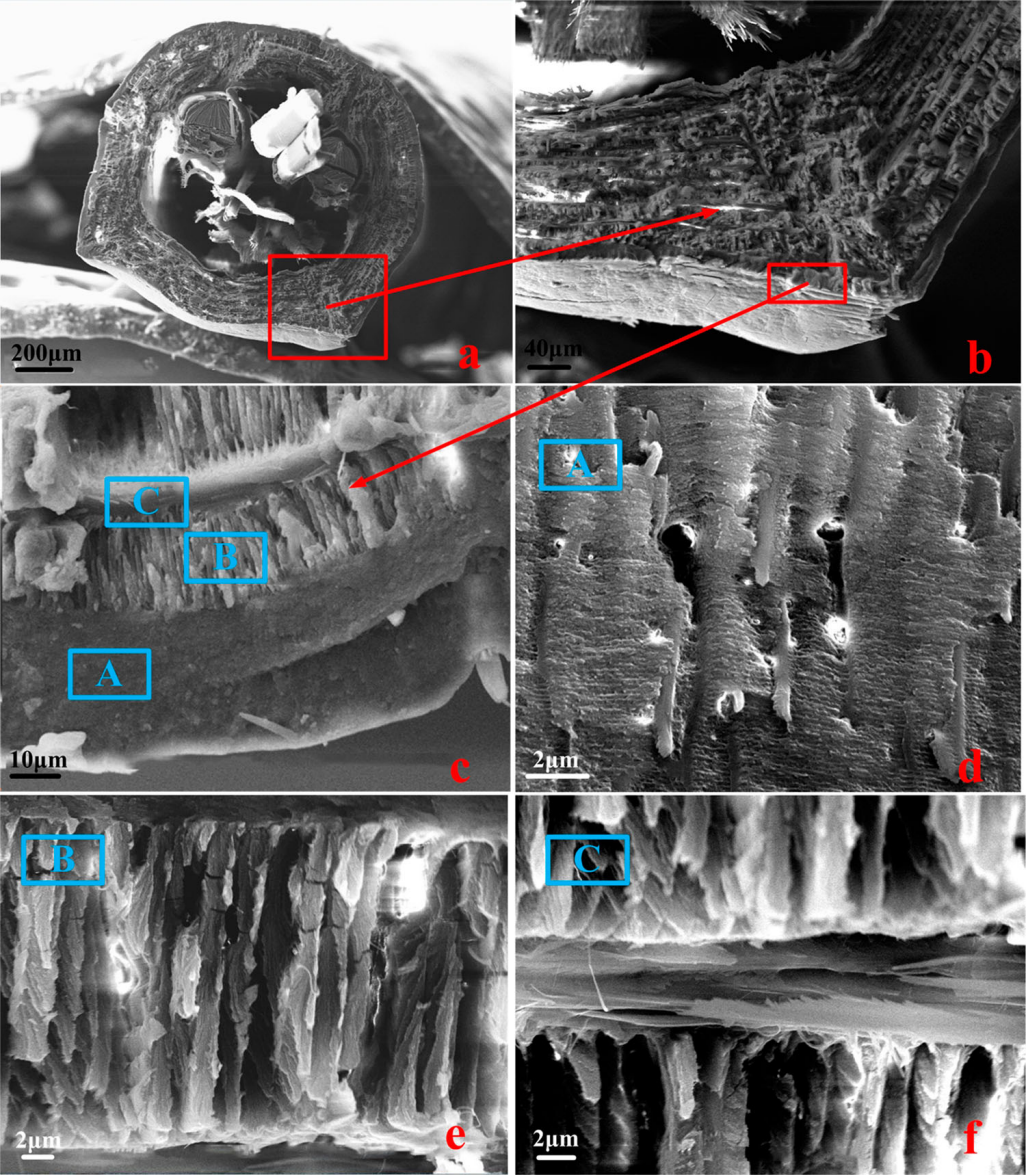
Cross section of the rostrum in *C. longimanus*. (a) Cross section of the rostrum; (b) Details in an enlarged view of the portion of Figure 3(a) that is indicated by the arrow; (c) multilayer structure composed of exocuticle layer (marked A) and endocuticle layer. Endocuticle layer is composed of the radial fibers (marked B) and the circumferential fibers (marked C); (d) details in an enlarged view of surface for exocuticle layer; (e) details in the radial fibers; (f) details in the circumferential fibers.

The outstanding mechanical properties of the rostrum are closely related to the macro- and microstructure. The rostrum connects the mouth and the head of the weevil, and bears a huge force and friction when it sucks on bamboo. It is a hollow multilayer cylindrical structure with a cross section as shown in Figure 3(a,b). The cuticle of the rostrum consists of exocuticle layer (EXO) and endocuticle layer (END); the endocuticle layer constitutes the thickest region. Endocuticle layer comprises the radial fibers (marked B) and the circumferential fibers (marked C), and the structure details can be seen in Figure 3(d–f). It is showed that exocuticle layer is a rigid external shell. Radial fibers and the circumferential fibers have many layers and are stacked alternately. Moreover, every layer of radial fibers consists of many flakelets that are mineralized materials and almost perpendicular to the layers of circumferential fibers. The thickness of layers for radial fibers decreases from the outside in. Circumferential fibers are convolved along the rostrum axis. The thickness of each layer for circumferential fibers is almost equal. These fibers connect with each other and keep a certain gap, playing a supporting role. These special fiber layer structures ensure that the rostrum lightweight, higher strength and higher stiffness. In addition, the rostrum of this weevil has a non-smooth surface (Figure 3(c,d)), which plays an important role in reducing adhesion and frictional resistance while drilling holes in bamboo.

### 3.2. Composition of the rostrum in *C. longimanus*

**Figure 4.**
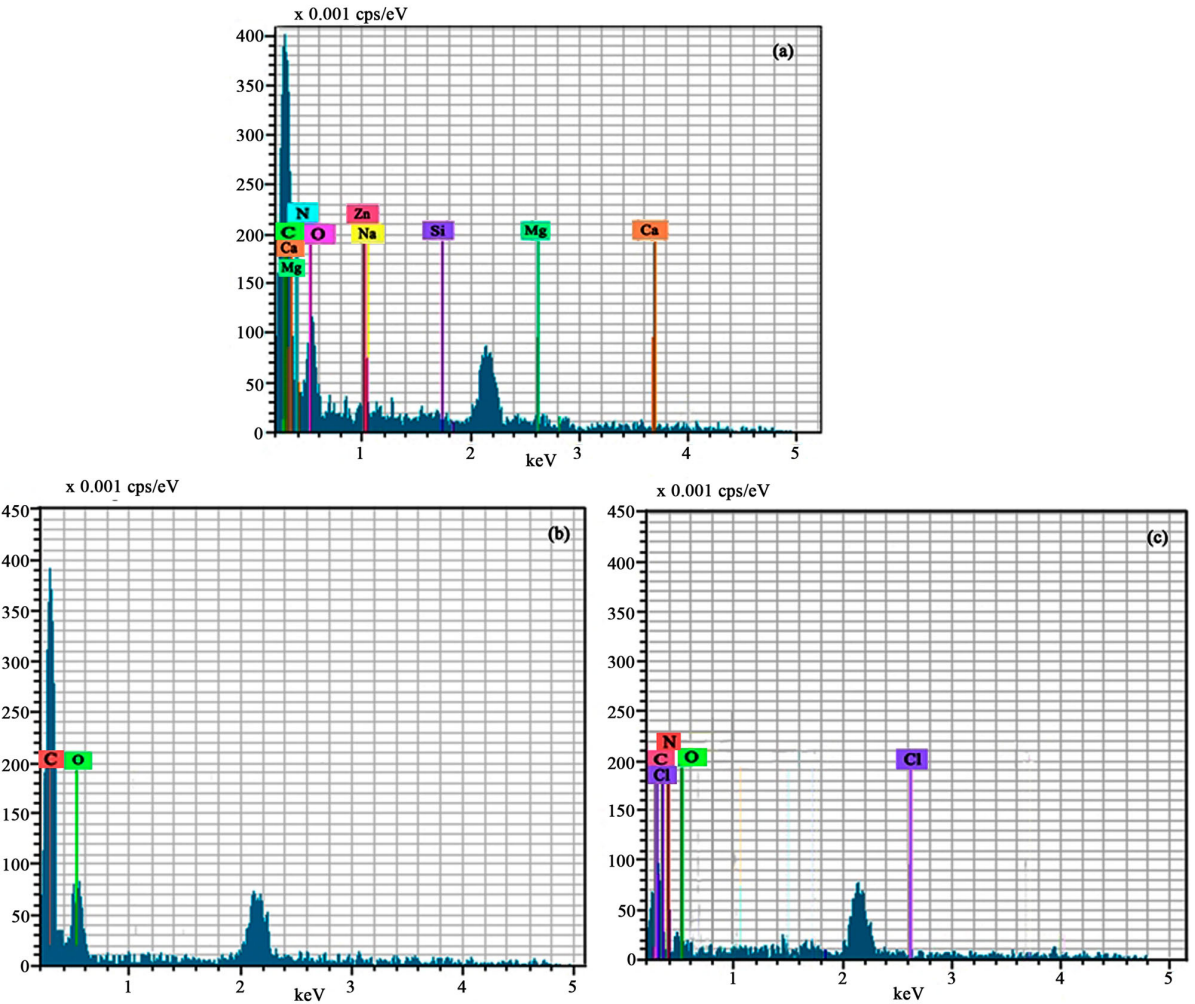
Energy spectrum diagram of different parts in the rostrum. (a) representative energy spectrum diagram of exocuticle layer; (b) representative energy spectrum diagram of radial fibers; (c) representative energy spectrum diagram of circumferential fibers.

The composition of exocuticle layer, radial fibers and circumferential fibers for the rostrum was obtained by EDS analysis. The energy spectrum diagram and elements table were shown in Figure 4. Exocuticle layer of the rostrum was mainly made of chitin, which includes C, H, N, O, metal elements and microelements, such as Mg, Si, Zn, Ca and Na (Figure 4(a)). The elements Mg and Si make the microstructure denser and harder, and help to maintain the shape of the rostrum. Radial fibers in the rostrum only consists of C, O, H elements, and the ratio of carbon to oxygen is about 61:39 (Figure 4(b)). From the energy spectrum diagram, it can be seen that radial fibers are composed of lipoid or saccharide with no protein and inorganic salts. It consists of many flakelets in ordered arrangement and there is a gap between adjacent two flakelets. The mechanical test results show that this structure can not only improve its distortion-resistant ability, but also help to the energy storage and release. The major components of circumferential fibers are cellulose and proteins, which includes several chemical elements, such as C, H, O, N, Cl, etc. (Figure 4(c)).

### 3.3. Mechanical properties of the weevil’s rostrum

**Figure 5.**
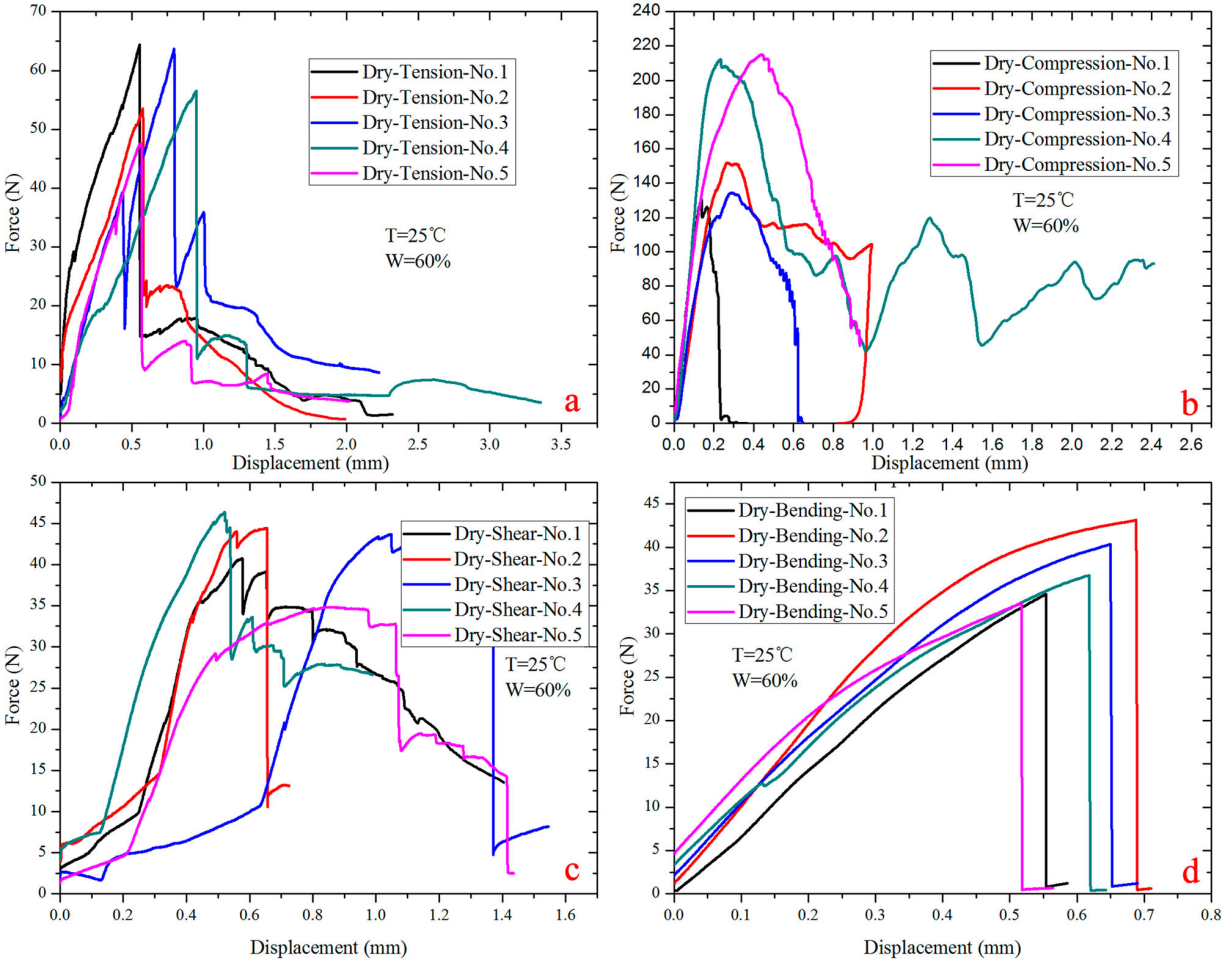
Mechanical properties of the ‘dry’ rostrum in *Cyrtotrachelus longimanus*; (a) tension force–displacement curves; (b) compressive force–displacement curves; (c) shear force–displacement curves; (d) bending force–displacement curves.

**Figure 6.**
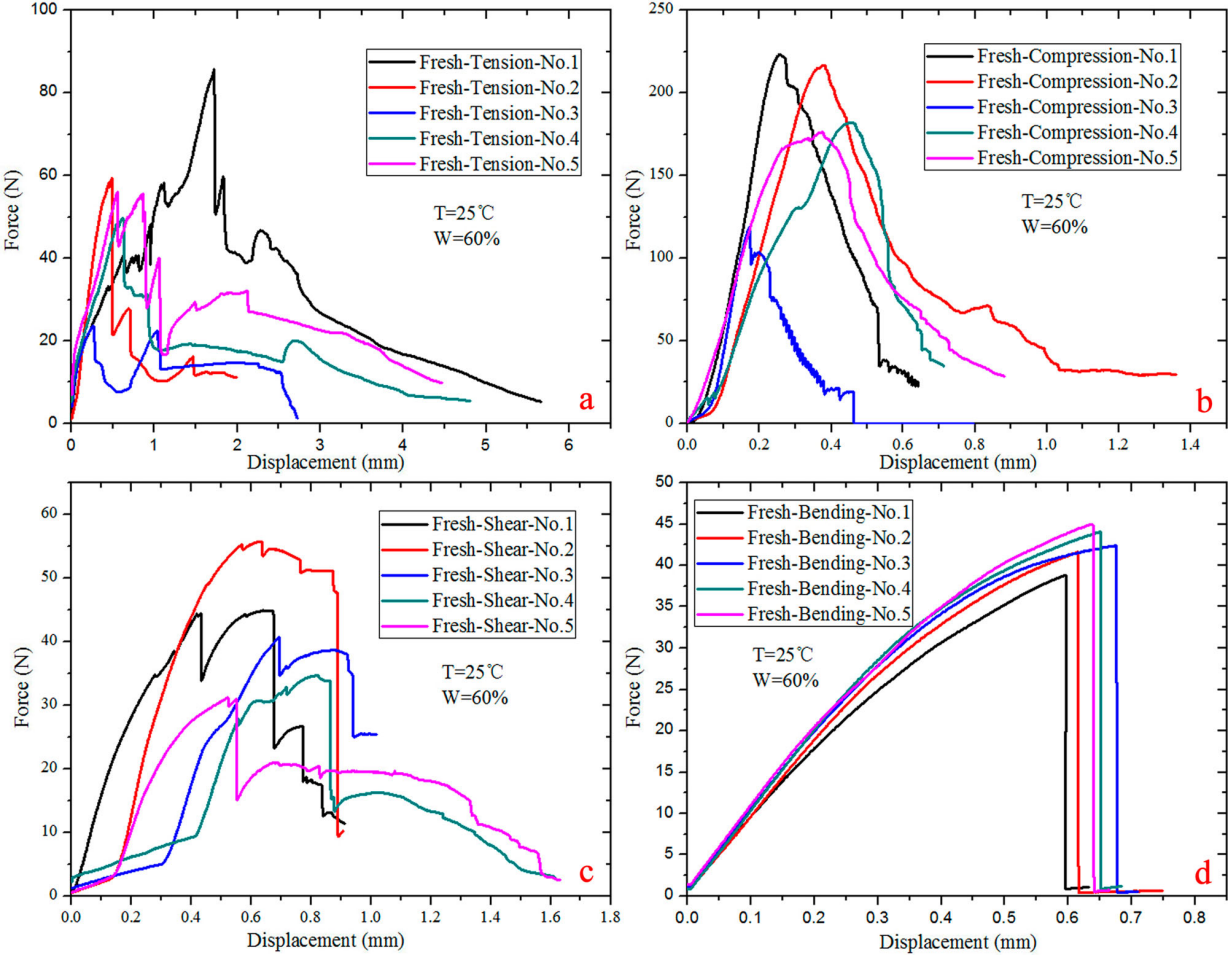
Mechanical properties of the ‘fresh’ rostrum in *Cyrtotrachelus longimanus*; (a) tension force–displacement curves; (b) compressive force–displacement curves; (c) shear force–displacement curves; (d) bending force–displacement curves.

The force–displacement curves of the rostrum materials under different kinds of load type were shown in Figures 5 and 6. It can be seen that the curves are almost linear in the elastic region, and fracture happened after a brief yield deformation, demonstrating the rostrum is mainly elastic deformation. According to the experimental results, mechanical properties of ‘dry’ and ‘fresh’ rostrum were calculated and the data are summarized in Table 1, which shows no clear difference between the dry samples and fresh ones. At the same time, the cross-section structure shows that the rostrum in *C. longimanus* is not homogeneous solid material, but a hollow porous composite material, which has excellent mechanical properties.

**Table 1.** Mechanical properties of rostrum.

	Dry samples (*n* = 20)	Fresh samples (*n* = 20)
Tensile strength (MPa)	20.52 ± 2.44	21.88 ± 4.64
Compressive strength (MPa)	60.7 ± 12.51	69.08 ± 4.53
Shear strength (MPa)	9.04 ± 0.88	9.42 ± 0.701
Bending strength (MPa)	189.62 ± 17.34	184.6 ± 13.02
Specific tensile strength (kN•m/kg)	26.31 ± 3.13	28.05 ± 5.95
Specific compressive strength (kN•m/kg)	77.82 ± 16.04	88.56 ± 5.81
Specific tensile stiffness (N/mm/(g/cm^3^))	110.38 ± 26.28	113.12 ± 39.26
Specific compressive stiffness (N/mm/(g/cm^3^))	643.13 ± 211.22	618.17 ± 190.28

## 4. Discussion

As stated previously, the outstanding structure of biological materials is a result of evolution (Chen et al. 2014). The specimen is a member of the beetle family *Curculionidae*, usually called weevils. In fact, it’s called this just because the feature of this insect is the rostrum, which extends from the head and bears the chewing mouthparts. From the biomechanical point of view, the macro-/microstructures of rostrum are of significance to the lightweight structure and materials.

In this paper, the fine structure of the rostrum for *C. longimanus* was presented for the first time. The rostrum is a rigid structure in the sense that it has no articulations or joints (Singh et al. 2016). The SEM of its cross section (Figure 3(a)) shows that the rostrum is a hollow multilayer composite structure, whose outer layers are loosely arranged and inner layers are density aligned. We suppose that this is related to load conditions when the weevil chews the bamboo shoots, and also is the result of evolution by nature. In addition, a special double helix can be found in Figure 3(c), the neighboring layers for both radial fibers and circumferential fibers in the endocuticle layer of the rostrum were aligned at 10°–20°, and radial fibers and circumferential fibers were aligned by 80°–90°. The fiber layers overlap each other and rotate a certain angle. This structure has excellent mechanical properties, high-specific strength and high-specific stiffness. The similar double-helix structure also can be seen in bamboo fibers (Ray et al. 2005; Tan et al. 2011; Wang et al. 2013; Huang et al. 2016; Zou 2016) and the shell of cicada and the scarab (Chen & Wu 2006; Rao 2012; Chandran et al. 2016).

The rostrum of *C. longimanus* has anisotropic properties. The radial fibers in the endocuticle layer have a reinforcement effect on the mechanical properties of the rostrum, which supporting and strengthening the rostrum. The experimental results also show that the compression strength of rostrum is three times as the tensile strength, demonstrating the compression ability of rostrum is far greater than its tensile ability. On the other hand, according to Table 1, there is no obvious mechanical difference between the ‘fresh’ rostrum samples and dried ones.

Being very light, the compressive specific strength of ‘dry’ samples were 77.82 ± 16.04 kN m/kg, and the ‘fresh’ samples were 88.56 ± 5.81 kN m/kg. The specific tensile stiffness and specific compressive stiffness of ‘dry’ samples were 110.38 ± 26.28 (N/mm/(g/cm^3^)) and 643.13 ± 211.22 (N/mm/(g/cm^3^)), and the values of ‘fresh’ samples were 113.12 ± 39.26 (N/mm/(g/cm^3^)) and 618.17 ± 190.28 (N/mm/(g/cm^3^)). For compressive specific strength of the rostrum, it is almost the same order as that of engineering alloys, for example, 304 stainless steel 65 kN m/kg (Wang & Meyers 2017). Therefore, the rostrum of the weevil can be an ideal model to inspire new lightweight structural design for the long tubular structure.

## 5. Conclusions

This paper presented early investigation of morphology, composition and mechanical properties of the rostrum in the *Cyrtotrachelus longimanus*. SEM was employed to observe the morphological features of the weevil’s rostrum. The results showed that the rostrum is a lightweight multilayer cylindrical structure, which consists of exocuticle layer and dense endocuticle layers. Radial fibers and circumferential fibers in endocuticle layers are stacked alternately, which can maximally save materials at the same time keep its excellent mechanical properties. Composition analysis of the weevil’s rostrum shows that some metal elements and microelements are contained in the rostrum, which contribute to its mechanical performance. The mechanical tests demonstrated its compressive specific strength almost equal to that of metal alloy stainless steel. Therefore, our works provide an ideal bionic model to design a lightweight long tubular structure.

## 6. Geolocation information

The field of my research involved morphology, lightweight biomaterials, biomimetic structural design and bionic engineering.

## References

[CIT0001] Andrew JM, Singh SS, Chawla N. 2016 A multilayer micromechanical model of the cuticle of *Curculio longinasus Chittenden*, 1927 (Coleoptera: Curculionidae). J Struct Biol. 195:139–158. doi: 10.1016/j.jsb.2016.05.007 27189867

[CIT0002] Chandran R, Williams L, Hung A. 2016 SEM characterization of anatomical variation in chitin organization in insect and arthropod cuticles. Micron. 82:74–85. doi: 10.1016/j.micron.2015.12.010 26774746

[CIT0003] Chen B, Wu XY. 2006 Analysis of abnormal fiber shape of Chafer cuticle. J Mater Sci Eng. 24:683–686. Chinese.

[CIT0004] Chen F, Wang W, Wang X. 2005 Hazard and prevention of *Cyrtotrachelus bugueti*. Plant Prot. 31:89–90. Chinese.

[CIT0005] Chen Q, Shi Q, Gorb SN. 2014 A multiscale study on the structural and mechanical properties of the Luffa sponge from Luffa cylindrica plant. J Biomech. 47:1332–1339. doi: 10.1016/j.jbiomech.2014.02.010 24636532

[CIT0006] Dai Z, Yang Z. 2010 Macro-/micro-structures of elytra, mechanical properties of the biomaterial and the coupling strength between Elytra in beetles. J Bionic Eng. 7:6–12. doi: 10.1016/S1672-6529(09)60187-6

[CIT0007] Davis SR. 2011 Rostrum structure and development in the rice weevil *Sitophilus oryzae* (Coleoptera: Curculionoidea: Dryophthoridae). Arthropod Struct Dev. 40:549–558. doi: 10.1016/j.asd.2011.06.002 21978823

[CIT0008] Huang Y, Fei B, Wei P. 2016 Mechanical properties of bamboo fiber cell walls during the culm development by nanoindentation. Ind Crop Prod. 92:102–108. doi: 10.1016/j.indcrop.2016.07.037

[CIT0009] Guo C, Li D, Lu ZY, Zhu CS, Dai ZD. 2014 Mechanical properties of a novel, lightweight structure inspired by beetle’s elytra. Chin Sci Bull. 59:3341–3347. doi: 10.1007/s11434-014-0384-5

[CIT0010] Guo C, Song WW, Dai ZD. 2012 Structural design inspired by beetle elytra and its mechanical properties. Chin Sci Bull. 57:941–947. doi: 10.1007/s11434-011-4956-3

[CIT0011] Li T, Xu X, Zhou Z, Gao Z, Deng G, Xiao T. 2000 A preliminary study on the biological characteristics of *Cyrtotrachelus buqueti* Gu'erin-M'eneville. J Sichuan Forest Sci Tech. 3:49–51. Chinese.

[CIT0012] Moon MJ. 2015 Microstructure of mandibulate mouthparts in the greater rice weevil, *Sitophilus zeamais* (Coleoptera: Curculionidae). Ento Res. 45:9–15. doi: 10.1111/1748-5967.12086

[CIT0013] Paine TD, Raffa KF, Harrington TC. 1997 Interactions among scolytid bark beetles, their associated fungi, and live host conifer. Annul Rev Entomol. 42:179–206. doi: 10.1146/annurev.ento.42.1.179 15012312

[CIT0014] Patek SN, Rosario MV, Taylor JR. 2013 Comparative spring mechanics in mantis shrimp. J Exp Bio. 216:1317–1329. doi: 10.1242/jeb.078998 23239886

[CIT0015] Porter MM, Mckittrick J, Meyers MA. 2013 Biomimetic materials by freeze casting. JOM. 65:720–727. doi: 10.1007/s11837-013-0606-3

[CIT0016] Rambo CR, Müller FA, Müller L. 2006 Biomimetic apatite coating on biomorphous alumina scaffolds. Mat Sci Eng C. 26:92–99. doi: 10.1016/j.msec.2005.06.003

[CIT0017] Rao R. 2012 Research progress on bionics based on morphological structure of insect (exoskeleton and wings). Mod Agr Sci Technol. 18:266–268.

[CIT0018] Ray AK, Mondal S, Das SK, Ramachandrarao P. 2005 Bamboo – a functionally graded composite correlation between microstructure and mechanical strength. J Mater Sci. 40:5249–5253. doi: 10.1007/s10853-005-4419-9

[CIT0019] Singh SS, Jansen MA, Franz NM. 2016 Microstructure and nanoindentation of the rostrum of *Curculio longinasus*, chittenden, 1927(Coleoptera:Curculionidae). Mater Charact. 118:206–211. doi: 10.1016/j.matchar.2016.05.022

[CIT0020] Sven B, Farid D, Ding L. 2015 Bio-inspired design to support reduced energy consumption via the ‘light weighting’ of machine system elements. Int J Mod Optimiz. 5:82–89. doi: 10.7763/IJMO.2015.V5.441

[CIT0021] Tabunoki H, Gorman MJ, Dittmer NT. 2016 Superoxide dismutase 2 knockdown leads to defects in locomotor activity, sensitivity to paraquat, and increased cuticle pigmentation in *Tribolium castaneum*. Sci Rep. 6:29583. doi: 10.1038/srep29583 27387523 PMC4937408

[CIT0022] Tan T, Rahbar N., Allameh SM. 2011 Mechanical properties of functionally graded hierarchical bamboo structures. Acta Biomater. 7:3796–3803. doi: 10.1016/j.actbio.2011.06.008 21704742

[CIT0023] Vincent JF, Wegst UG. 2004 Design and mechanical properties of insect cuticle. Arthropod Struct Dev. 33:187–199. doi: 10.1016/j.asd.2004.05.006 18089034

[CIT0024] Wang B, Meyers MA. 2017 Seagull feather shaft: correlation between structure and mechanical response. Acta Biomater. 48:270–288.

[CIT0025] Wang F, Shao Z, Wu Y. 2013 Mode II interlaminar fracture properties of Moso bamboo. Compos Part B: Eng. 44:242–247. doi: 10.1016/j.compositesb.2012.05.035

[CIT0026] Wang S, Yang Y, Liu C, Liang Z, Wan D. 2009 Morphological anatomy of reproductive system of *Cyrtotrachelus buqueti guer*. Sichuan J Zool. 28:79-81. Chinese.

[CIT0027] Wang W, Chen F, Wang X. 2005 Reproductive behavior of *Cyrtotrachelus bugueti*. Sichuan J Zool. 24:540–541. Chinese.

[CIT0028] Yang ZX, Dai ZD, Guo C. 2010 Morphology and mechanical properties of *Cybister elytra*. Chin Sci Bull. 55:771–776. doi: 10.1007/s11434-009-0363-4

[CIT0029] Yi WK, Du B. 2003 Aerospace manufacturing technology. Beijing: China Astronautic Publishing House.

[CIT0030] Zou M, Xu S, Wei C. 2016 A bionic method for the crashworthiness design of thin-walled structures inspired by bamboo. Thin Wall Struct. 101:222–230. doi: 10.1016/j.tws.2015.12.023

